# Cost comparison of minimally invasive transforaminal lumbar interbody fusion: experience from Nigeria and the United States

**DOI:** 10.3389/fpubh.2026.1790794

**Published:** 2026-09-28

**Authors:** Oluwafemi F. Owagbemi, Temitayo O. Ayantayo, Ugochukwu C. Odekpe, Chidera E. Uzuegbunam, Oloruntoba E. Ogunfolaji, Olawale A. R. Sulaiman

**Affiliations:** 1 RNZ Clinic, Lagos, Nigeria; 2 Neuroscience and Mental Health Institute, University of Alberta, Edmonton, AB, Canada

**Keywords:** cost-effective care, developing country, direct cost, global neurosurgery, medical tourism, minimally invasive spine surgery, minimally invasive transforaminal lumbar interbody fusion, postoperative outcome

## Abstract

**Introduction:**

The total cost of patient care for minimally invasive spine surgery is generally lower than for open spine surgeries, primarily due to shorter length of hospital stay (LOS), shorter surgery duration (SDn), lower perioperative morbidity, and reduced narcotic pain medication use. Due to prohibitive costs, neither surgical approach is readily accessible in developing countries. Objectives—to report our experience with minimally invasive transforaminal lumbar interbody fusion (MIS-TLIF) in Nigeria, a lower-middle-income country, and to compare it with data from our prior practice in the United States (U. S.).

**Methods:**

An observational, comparative analysis was conducted between two retrospective cohorts of MIS-TLIF patients in Nigeria and the U. S. Demographic data, perioperative parameters (SDn, estimated blood loss [EBL], and LOS), perioperative surgical site complications, and patient-reported outcomes (Numeric Rating Scale [NRS] and Oswestry Disability Index [ODI]) for patients who underwent MIS-TLIF for degenerative spine disease from June 2018 to May 2022 in the Nigerian practice were retrieved from the database. Minimal clinically important differences (MCID) in patient-reported outcomes (PROs) were defined as a two-point change on the NRS and a 5.9–20-point difference on the ODI. The hospital’s accounts section provided the direct costs, which were converted from Nigerian Naira to U. S. dollars. Data from both cohorts, which had similar surgical indications, the same lead surgeon, and similar operative techniques and perioperative management, were compared.

**Results:**

The median age of the 65 patients was 61 years; 37 were female. The median SDn, EBL, and LOS were 190 min, 100 mL, and 3 days, respectively. Compared with the MIS-TLIF subgroup in the previous cohort, this cohort had higher mean SDn (186.2 versus 161.0 min, *p* < 0.001), EBL (104.8 versus 95.0 mL, *p* = 0.264), and LOS (4.1 versus 3.6 days, *p* = 0.558), as well as higher perioperative surgical site complication rates (9.2% versus 7.0%, *p* = 0.749). As in the U. S. cohort, PROs showed statistically significant improvement and met the MCID from baseline to the 12-month follow-up. The mean cost was lower in this study ($9,530 versus $20,960, *p* < 0.001).

**Conclusion:**

MIS-TLIF direct costs are lower in our Nigerian practice than in a developed country, despite outcomes that are mostly statistically similar.

## Introduction

Spine surgery is expensive (1, 2); however, with spinal trauma and degenerative spine disease (DSD) as two of the leading conditions requiring medical care worldwide (3–6), spine surgery remains a necessity. This is especially true given the increasing global life expectancy (7), and the rising prevalence of spinal conditions in the older adults and in the aging spine (8). Life expectancy in Nigeria, though lower than the global average, is also increasing (9), and degenerative spine disease is increasingly diagnosed in the country (10–13). The need to expand spine surgery services in Nigeria is therefore notable (14). Open instrumented and non-instrumented spine surgeries have been available in the country for decades (10, 14–17), but minimally invasive spine surgery (MISS) became available only recently, and we recently published the country’s first series (18).

The benefits of MISS over open approaches are well established (19), and the rationale for introducing MISS in developing countries has been discussed (18), especially in countries where open spine surgeries are already performed, such as Nigeria. An advantage of MISS over open spine surgery is that the total cost of patient care is generally lower, primarily due to shorter hospital stays, shorter operative times, lower perioperative morbidity, and reduced use of narcotic pain medication (20, 21). However, prohibitive costs make neither surgical approach readily accessible in lower-middle-income countries (LMICs) such as Nigeria (22), where MISS remains nascent (18).

Our objectives are to report our experience with perioperative parameters, perioperative surgical complications, patient-reported outcomes, and direct hospital costs for minimally invasive transforaminal lumbar interbody fusion (MIS-TLIF), a type of MISS, in our practice in Nigeria, and to compare these data with those from our prior practice in the United States (U. S.). We hypothesized that perioperative parameters and surgical site complication rates would not differ between cohorts, and that direct hospital costs would be lower in the Nigerian cohort.

## Materials and methods

### Study design

This study is an observational, comparative analysis of two MIS-TLIF cohorts: one from our Nigerian practice and the other from our U. S. practice, which serves as a historical cohort.

### Settings

In the United Nations Development Program’s 2021/22 Human Development Report, Nigeria ranked 163rd, and the United States ranked 21st on the Human Development Index (HDI) among 191 nations. Nigeria was thus classified as a low-human-development nation, and the United States as a very high-human-development nation (23). The World Bank’s 2020 Human Capital Index (HCI) tells a similar story, with values of 0.36 and 0.70 for Nigeria and the United States, respectively (the HCI ranges from 0 to 1) (24). These metrics show a stark difference in development levels between the two countries. Lagos, where the Nigerian practice is located, was reported to have the highest HDI in Nigeria in 2022, at 0.721, while the United States practice was in Louisiana, which had an HDI of 0.881 in the same year (25, 26).

Nigeria has a population of approximately 220 million (27). According to the Multidimensional Poverty Index, which “captures the multiple deprivations that people in developing countries face in their health, education and standard of living,” Nigeria was reported to have 40.1% of its population living below the national poverty line and 39.1% living below the international poverty line of $1.90 a day (23), whereas the U. S. is a high-income country (28). Poverty in Nigeria is further worsened by limited access to healthcare, linked to poor social health insurance, which was recently reported to cover less than 5% of the population, with most people paying out-of-pocket for healthcare (29).

The Nigerian practice is at RNZ Clinic, Victoria Island, Lagos, Nigeria, a private clinic, and the surgeries were performed at a private hospital (Euracare Multi-Specialist Hospital, Victoria Island, Lagos, Nigeria) with a single operating room. The U. S. hospital, Ochsner Medical Center, New Orleans, Louisiana, is also private. Operating rooms in both hospitals were equipped with a Jackson table, a power drill, electrocautery, an operating microscope, and a fluoroscopy unit, all of which were standard for MIS-TLIF. In both settings, a 22 mm tubular retractor (METRx System, Medtronic, Memphis, TN, USA) was used to maintain retraction during the MIS-TLIF procedures, and percutaneous pedicle screw instrumentation was performed (30). Modern hemostatic agents used in the U. S. cohort were not available for surgeries in Nigeria.

### Participants

The Nigerian cohort comprised patients who underwent MIS-TLIF for degenerative lumbar spondylolisthesis, with or without foraminal stenosis, at our practice from June 2018 to May 2022. The diagnosis of these pathologies was based on clinical features of mechanical low back pain, with or without radicular leg pain, and was corroborated by magnetic resonance imaging (MRI) findings. Surgery was recommended after 6 weeks of nonoperative treatment (oral medication and physical therapy, with or without an epidural steroid injection) failed. No patient who met the inclusion criteria was excluded from the study. The rationale for selecting MIS-TLIF over open-TLIF is discussed in our previous MISS report (18).

All MIS-TLIF procedures were performed by the senior author, with some cases assisted by co-authors, using a technique similar to that described in a previous publication (30). All wounds were copiously irrigated and closed in layers, and all patients received similar postoperative care using standardized postoperative order sets. Across procedures, care was nearly identical for patients, who were referred for physical therapy two to 4 days postoperatively, as needed. This approach is also consistent with the senior author’s method in the United States (30).

### Variables, data sources, and measurement

Study data included patient demographics (age and sex), diagnoses, perioperative data (surgeries performed, number of spinal levels [NOL] operated on, surgery duration [SDn], estimated blood loss [EBL], and length of hospital stay [LOS]), perioperative surgical site complications, patient-reported outcomes (PROs), and direct hospital costs. The primary outcomes were the perioperative parameters (SDn, EBL, and LOS), perioperative surgical site complications, PROs, and costs.

We defined SDn as the duration, in minutes (min), from skin incision to final closure stitch, and EBL as blood loss during surgery, in milliliters (mL), estimated visually. These definitions are similar to those used by other workers (31–34) and were documented in hospital records by the operating room staff. LOS was defined as the duration from surgery to hospital discharge, recorded in days. SDn and EBL were obtained from operating room records, and LOS from patients’ admission records, for entry into a prospectively maintained study database. The observation of these outcome variables was limited to the admission period.

Perioperative surgical complications considered in the study included nerve injury, dura tear, pseudomeningocele, malpositioned implants, implant migration, and early surgical site infection (SSI) when identified by the surgeon. A nerve injury was diagnosed if it occurred during surgery or if the patient developed new postoperative weakness. A dura tear was diagnosed if it occurred intraoperatively or if cerebrospinal fluid (CSF) leakage was observed postoperatively. A pseudomeningocele was defined as a fluctuant swelling with an abnormal CSF collection outside the dura mater, as demonstrated on postoperative MRI. Implant malposition and migration were confirmed by postoperative computed tomography in patients with dermatomal leg pain. Wound evaluation was performed per practice protocol—as needed during admission, on the seventh postoperative day, and at 1 month postoperatively—with discharge instructions for signs of wound infection provided to the patient and caregivers. In accordance with the Centers for Disease Control and Prevention, USA, criteria, an early SSI was diagnosed if an infection occurred at the surgical site within 30 days of surgery (35). To focus the evaluation primarily on surgical technique and the operative environment, we limited the review of complications in this study to surgical site complications occurring within 30 days of surgery, as recorded in the database.

For patient-reported outcomes, we evaluated low back and leg pain using the 11-point Numeric Rating Scale (NRS) (36), which ranges from 0 (no pain) to 10 (worst pain imaginable), and disability on a 0–100% scale using the Oswestry Disability Index (ODI) (37, 38). We assessed these outcomes using self-administered questionnaires at baseline (preoperatively) and at one, six, and 12 months after surgery, looking for minimal clinically important differences (MCID), defined as a two-point change on the NRS and a 5.9–20-point difference on the ODI (39, 40).

Direct costs for the Nigerian cohort included surgical and anesthesia fees, medical supplies costs (including implants), and total charges from admission through discharge. These costs were provided by the hospital’s accounts section.

The data in this study were compared with those from a study published by the senior author, based on his prior practice in the U. S. The U. S. study reported perioperative parameters, surgical complications, and direct hospital costs for open- and MIS-TLIF, and we extracted the MIS-TLIF results from that article for our comparison. In the historical cohort, comparison data were presented as means, standard deviations (SDs), and proportions. Direct hospital cost data reported in the article were provided by the hospital accounting and billing office and included labor, medical supplies (including implants), and other costs, such as equipment and general/administrative costs, that are expensed across all hospital cost centers (30).

The costs in the Nigerian cohort were converted from Nigerian Naira (NGN) to U. S. Dollars (USD) for comparison with our previous cohort’s costs. We deliberately used conversion and inflation assumptions that biased the primary analysis against lower costs in the Nigerian setting. To achieve this, we converted Nigerian costs at the strongest official NGN-to-USD exchange rate observed during the study period (₦305.90/USD in July 2018), yielding the highest possible USD equivalent (41). We adjusted the U. S. cohort’s mean cost ($19,098; 2009–2012) for inflation over the shortest interval between the two studies (December 2012 to June 2018) using the U. S. Bureau of Labor Statistics (BLS) Consumer Price Index (CPI) inflation calculator to obtain a headline CPI. This yielded the lowest inflation-adjusted U. S. cost ($20,960) (42, 43). Any cost differential observed under this jointly conservative (Nigerian and U. S.) cost specification, therefore, represents a lower bound. Because a Nigerian patient seeking care abroad must purchase U. S. dollars to pay U. S. prices, we express the comparison as the percentage by which the U. S. cost exceeds the Nigerian cost, the quantity most relevant to that decision.

To assess the robustness of this finding, we conducted sensitivity analyses across alternative exchange-rate assumptions [the period-average official rate (41); the NAFEX/Investors’ & Exporters’ window rate (44); and the parallel-market rate (45)] and alternative inflation assumptions [adjustment to the Nigerian study midpoint and end; midpoint-to-midpoint comparison; and the BLS Medical Care CPI in place of the headline CPI (46)]. We also report a purchasing power parity (PPP) conversion of the Nigerian cost (47, 48).

The study’s continuous variables are age, SDn, EBL, LOS, and cost, and the PROs, although ordinal and discrete, were analyzed as continuous variables. Perioperative surgical site complication occurrence (the rate is a continuous variable) and sex are categorical variables, while NOL is an ordinal discrete variable. The independent variables are patients’ demographics, diagnostic data, and surgical procedures, while the dependent variables include SDn, EBL, LOS, perioperative surgical site complication rates, PROs, and cost.

The database was accessed on January 31, 2024, for the research. Patient consent was obtained for all procedures, and the Nigerian Institute of Medical Research’s Institutional Review Board granted ethical approval for the study, with a waiver of informed consent.

### Statistical analysis and study reporting

Statistical analyses were performed using jamovi version 2.7.31 (49) and R version 4.6.0 (50), with statistical significance set at a two-sided *p* ≤ 0.05 (51, 52). We evaluated the data using the Shapiro–Wilk test for normality and corroborated this with an assessment of asymmetry using D’Agostino’s skewness test (53). This informed our choice between means, with SDs and medians, with interquartile ranges (IQRs), as well as between parametric and nonparametric tests. Based on the normality and skewness assessments, we reported the cohort’s results as counts, percentages, and medians. A subanalysis was performed using the Kruskal-Wallis test for SDn, EBL, and LOS, and Fisher’s exact test for the perioperative surgical complication rate, with NOL as the grouping variable. Since the historical cohort averages were provided as means, we compared continuous data for which the mean, SD, and sample size were available (SDn, EBL, and LOS) using Welch’s two-sample t-test, which does not assume equal variances. Age and costs, for which the historical cohort provided only the mean, were compared using a one-sample t-test. Chi-squared or Fisher’s exact test was used to compare categorical data, as appropriate (30). Effect sizes (Cohen’s d), odds ratios, and 95% confidence intervals are reported.

Analysis of variables with ≤ 5% missing data that were assumed to be missing completely at random (MCAR) was performed using available-case analysis without imputation (54–58). Where the missing data exceeded 5%, Little’s test (59) was performed to assess MCAR, after which the following primary and sensitivity analyses were performed on the affected outcome variables.

The change in each affected outcome across observation points was modeled using a linear mixed-effects model for repeated measures, including a fixed effect for time (each observation time) and a random intercept for each patient, fitted by restricted maximum likelihood (60, 61). This likelihood-based method uses all available observations under the missing-at-random (MAR) assumption without imputation (62) and accommodates intermittent (non-monotone) missing-data patterns. The baseline observation was modeled as part of the outcome, so that patients without a preoperative score still contributed. Estimated marginal means at each timepoint and all pairwise between-timepoint comparisons (Tukey-adjusted) were obtained, and changes were interpreted relative to MCIDs of 2 points for the NRS and 5.9–20 points for the ODI (39, 40),

To assess robustness to a missing-not-at-random (MNAR) mechanism, a delta-adjusted multiple-imputation (tipping-point) analysis was performed (63, 64). Fifty imputed datasets were generated, and a progressively larger offset (delta) representing worse outcomes was added to the imputed postoperative values. The mixed model was then refitted and pooled across imputations using Rubin’s rules (65). The tipping point was the offset at which any improvement lost statistical significance or fell below the MCID. Further model details are provided in the Supplementary material.

The paired Wilcoxon signed-rank test with normal approximation was used to compare baseline PROs with follow-up values in a pairwise manner, serving as a potential complete-case corroboration of the primary analysis. This analysis was conducted without imputation. In addition, the median within-patient change (the effect) was reported (58).

We implemented the Strengthening the Reporting of Observational Studies in Epidemiology (STROBE) guideline in reporting this research (66).

## Results

A total of 65 patients underwent MIS-TLIF during the four-year study period. Some continuous variables (EBL, LOS, baseline NRS, cost, and PROs) were not normally distributed; however, further analysis showed that only EBL and LOS had statistically significant positive skewness (skewness = 2.27, z = 5.40, *p* < 0.001; skewness = 7.72, z = 9.57, *p* < 0.001). In contrast, the other variables showed no statistical evidence of asymmetry (Supplementary Table S1). Missingness was ≤ 5% for all variables except the PROs. The median age was 61 years (IQR 53–68 years). Most (37/65) were female. Forty MIS-TLIFs were performed for single-level disease, while the remainder involved two levels.

The median (IQR) values for SDn, EBL, and LOS were 190 (145–219) min, 100 (50–100) mL, and 3 (3) days, respectively, with a large LOS outlier of 60 days. Perioperative surgical complications included two dural tears, two malpositioned screws, one SSI, and one nerve injury, yielding a rate of 9.2% (6/65). Compared with one-level surgery, two-level surgery was associated with higher SDn (*p* < 0.001), EBL (*p* = 0.037), and LOS (*p* = 0.033), whereas perioperative surgical complication rates were similar (*p* = 0.668). Excluding the patient with the outlying LOS rendered the differences in EBL (*p* = 0.064) and LOS (*p* = 0.063) statistically insignificant.

Revision surgeries were performed on both patients with malpositioned screws to correct them. One patient with a dural tear and postoperative CSF leakage developed a deep SSI, leading to prolonged hospitalization. He required a second procedure for dural repair and a third for wound debridement and washout. The second dural tear and the nerve injury occurred in the same patient. The nerve injury resulted in a right-sided foot drop that improved with physiotherapy, while the dural tear was repaired during the same surgery without postoperative CSF leakage. One patient succumbed to a pulmonary embolism on the seventh postoperative day. She was a morbidly obese female who developed sudden, severe symptoms and rapid clinical deterioration.

Thirty-nine and 43 patients, respectively, completed their baseline NRS and ODI assessments. Follow-up evaluation rates at one, six, and 12 months were 41.0, 30.8, and 41% for NRS, and 37.2, 37.2, and 39.5% for ODI; thus, postoperative missingness was high for both outcomes. The pattern of missingness was non-monotone (intermittent), affecting 23 patients for ODI and 21 for NRS. Based on Little’s test, we did not reject the null hypothesis that data were missing completely at random for either outcome (NRS: χ^2^ = 27.2, df = 23, *p* = 0.25; ODI: χ^2^ = 22.6, df = 23, *p* = 0.49) (59). However, we assumed an MAR pattern for the primary analysis, given the limited power of Little’s test in the setting of high PRO missingness, as well as the occurrence of death-related dropout and possible perioperative complication-related dropout. We also examined departures from MAR using sensitivity analyses.

The mixed models included 83 NRS observations from 52 patients and 92 ODI observations from 55 patients. The effect of time on pain was significant (*F*(3, 69.5) = 35.2, *p* < 0.001; intraclass correlation = 0.17). Mean NRS fell from 7.97 (95% CI 7.40–8.55) at baseline to 3.99 (3.10–4.88) at 1 month and remained essentially stable thereafter (4.25 at 6 months, 3.93 at 12 months, Table 1). All three postoperative timepoints differed significantly from baseline (reductions of 3.7–4.0 points; all *p* < 0.001), whereas none of the Tukey-adjusted pairwise differences among postoperative timepoints were significant (all *p* ≥ 0.96; Table 2). Clinically important pain relief, exceeding the 2-point NRS (MCID), was thus achieved by 1 month and sustained through 12 months. There was also a significant overall effect of time on disability (*F*(3, 56.8) = 15.7, *p* < 0.001; intraclass correlation = 0.47). Estimated mean ODI declined progressively from 55.6 (95% CI 50.2–61.1) at baseline to 44.7 (36.5–53.0) at 1 month, 37.4 (29.1–45.7) at 6 months, and 27.0 (18.9–35.1) at 12 months (Table 1). In Tukey-adjusted pairwise comparisons (Table 2), disability was significantly lower than baseline at 6 months (−18.2 points, *p* < 0.001) and at 12 months (−28.6 points, *p* < 0.001), and improved further between 1 month and 12 months (−17.7 points, *p* = 0.007). The baseline-to-one-month reduction (−10.9 points, *p* = 0.075) and the differences between adjacent later visits did not reach significance after correction. The estimated 12-month improvement (28.6 points) exceeded the upper bound of the ODI MCID range (20 points).

**Table 1:** Estimated marginal means (95% CI) by time point from the mixed models. NRS on 0–10 scale; ODI on 0–100 scale.

Timepoint	NRS, mean (95% CI)	ODI, mean (95% CI)
Baseline	7.97 (7.40–8.55)	55.6 (50.2–61.1)
One month	3.99 (3.10–4.88)	44.7 (36.5–53.0)
Six months	4.25 (3.22–5.27)	37.4 (29.1–45.7)
12 months	3.93 (3.04–4.82)	27.0 (18.9–35.1)

**Table 2:** Pairwise between-timepoint contrasts (Tukey-adjusted).

Comparison	NRS diff (p)	ODI diff (p)
One month vs. baseline	−3.98 (<0.001)	−10.9 (0.075)
Six months vs. baseline	−3.72 (<0.001)	−18.2 (<0.001)
12 months vs. baseline	−4.05 (<0.001)	−28.6 (<0.001)
Six months vs. one month	+0.26 (0.981)	−7.4 (0.518)
12 months vs. one month	−0.07 (1.000)	−17.7 (0.007)

Values are the difference (later minus earlier); negative = improvement.

Under a progressively pessimistic delta adjustment for sensitivity to MAR departures (Supplementary Table S2), the one-month and 12-month baseline reductions remained significant across the entire range examined (through delta = 3), while the six-month reduction remained significant to an offset of 2 points and was borderline at 3 points. The 12-month disability improvement remained statistically significant across the entire range tested, attenuating from −30.4 points (delta = 0) to −15.6 points at an extreme offset of 20 points (*p* = 0.001) and staying above the lower ODI MCID bound (5.9 points) throughout. The six-month disability improvement remained significant to an offset of approximately 10 points, whereas the baseline-to-one-month improvement was fragile, losing significance once the offset reached about 5 points. Therefore, the principal findings, which are substantial improvements in pain by 1 month and in disability by 1 year from surgery, held under strong assumptions that patients who missed their follow-up visits fared worse than observed.

The median (IQR) NRS scores at baseline, 1 month, 6 months, and 12 months were 8.0 (7.0–9.0), 4.0 (3.0–5.0), 4.5 (2.5–6.3), and 4.0 (2.8–6.0), respectively, while the median (IQR) ODI scores at baseline, 1 month, 6 months, and 12 months were 56.0% (43.0–68.0%), 47.0% (25.0–58.5%), 40.0% (27.0–45.5%), and 24% (10.0–44.0%), respectively (Figures 1, 2). Pairwise comparisons of baseline and follow-up NRS scores for complete-case corroboration without imputation (58, 67) showed statistical differences at one, six, and 12 months (W = 55.0, median change = −4, *p* = 0.006; W = 21.0, median change = −5, *p* = 0.035; and W = 36.0, median change = −5, *p* = 0.014). For ODI, statistical differences were observed at six and 12 months; the one-month change did not reach significance (1 month: W = 64.0, median change = −13%, *p* = 0.052; 6 months: W = 66.0, median change = −26%, *p* = 0.004; and 12 months: W = 45, median change = −28%, *p* = 0.009). The median of differences met the criteria for minimal clinically important differences at all follow-up time points. These results are consistent with the mixed-model analysis.

**Figure 1:**
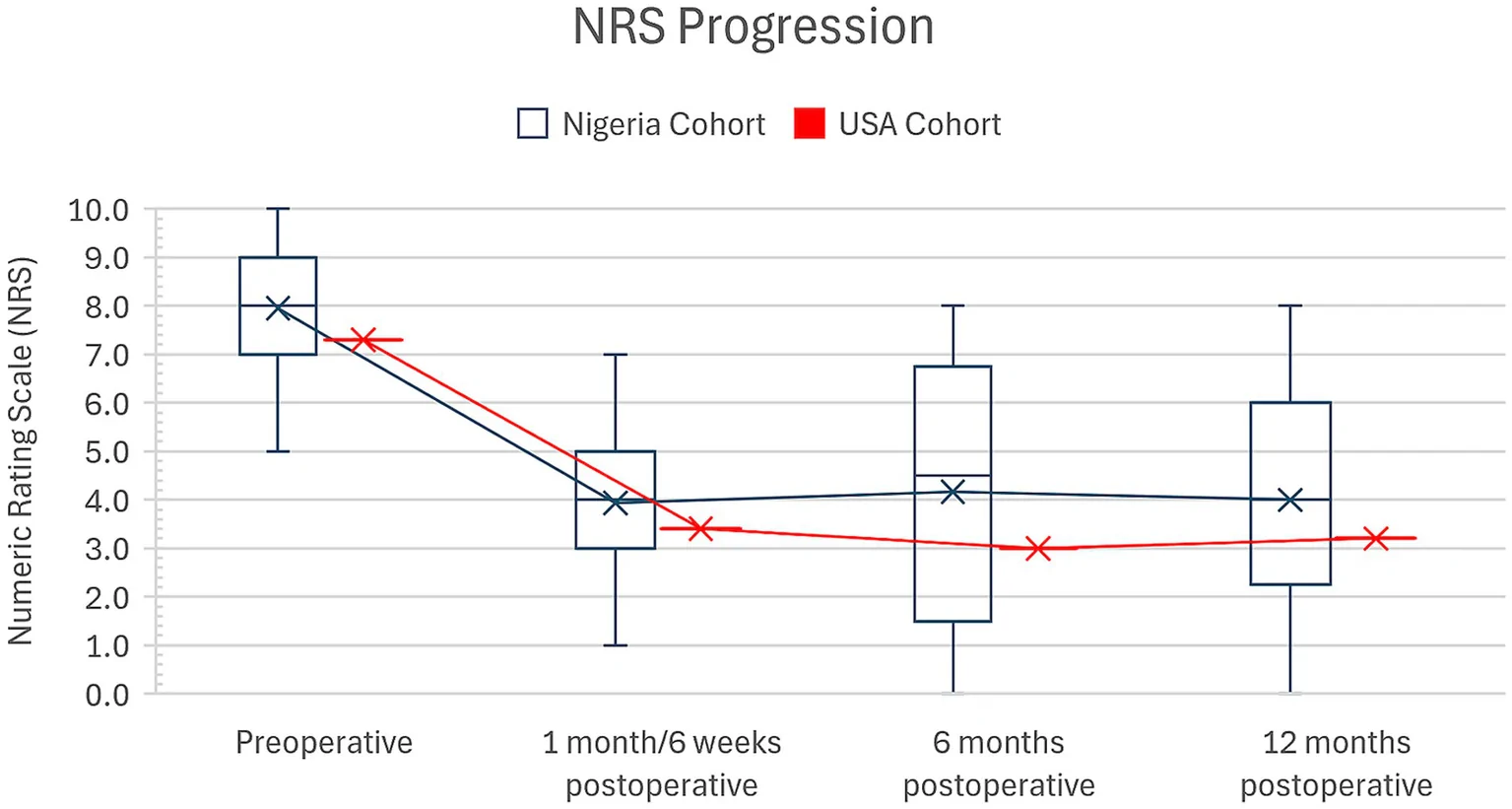
Progression of Numeric Rating Scale scores in both cohorts (box plots summarize the Nigerian cohort’s data, line graphs show the means of both cohorts [Nigerian—blue and US—red], and the first follow-up is 1 month in this study but 6 weeks in the previous study).

**Figure 2:**
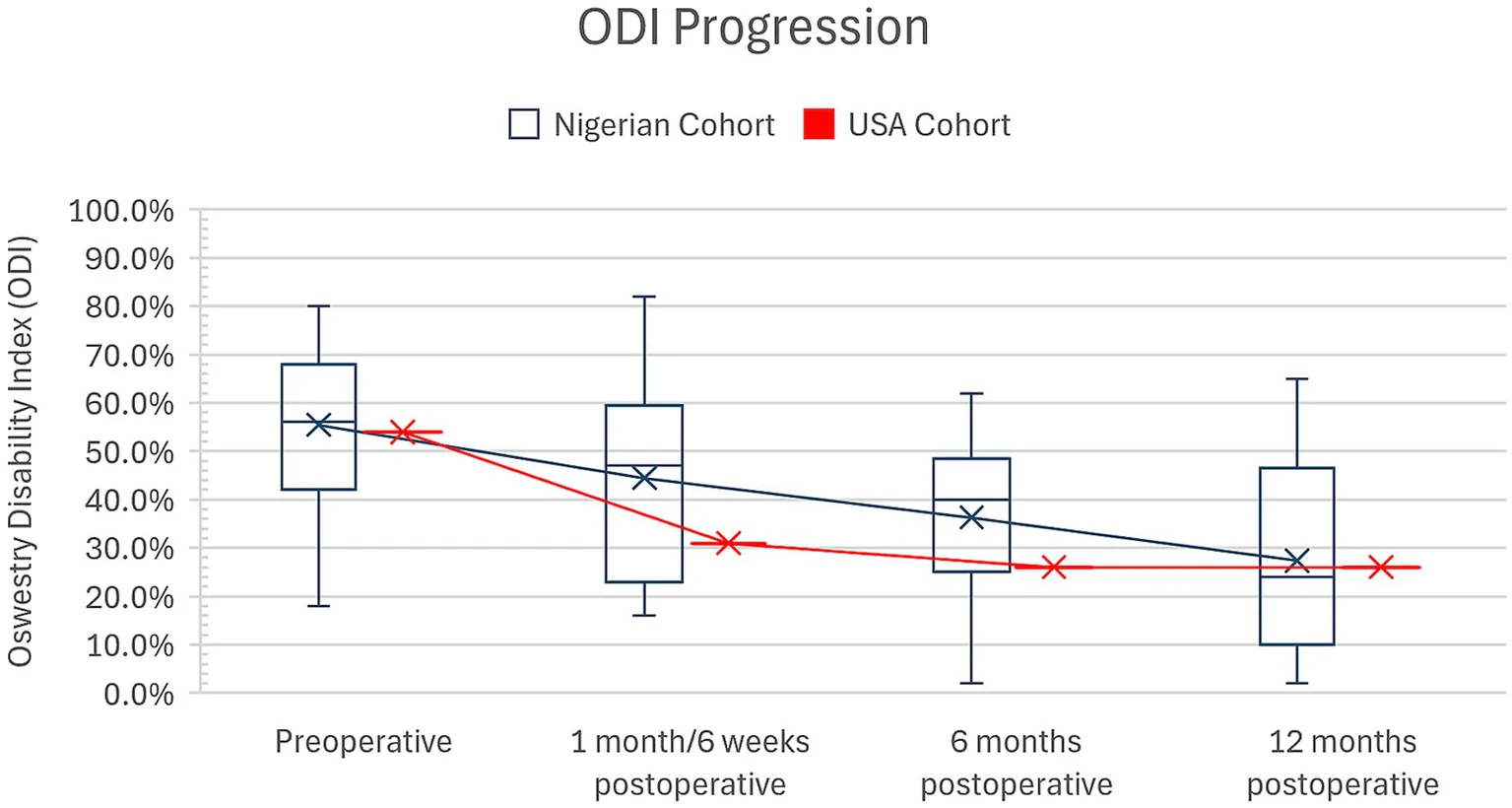
Progression of Oswestry Disability Index scores in both cohorts (box plots summarize the Nigerian cohort’s data, line graphs show the means of both cohorts [Nigerian—blue and US—red], and the first follow-up is 1 month in this study but 6 weeks in the previous study).

Using the primary (jointly conservative) specification, the inflation-adjusted U. S. cost of $20,960 exceeded the Nigerian cost ($9,530 from the 63 patients with cost data) by 119.9% (about 2.2-fold). This finding was robust across all 35 combinations of exchange-rate and inflation assumptions examined (Supplementary Table S3). Across officially sourced exchange rates, the U. S. cost exceeded the Nigerian cost by 100 to 251% (roughly 2.0–3.5×), and including an illustrative parallel-market conversion extended the range to 404% (up to ~5×). Converting each patient’s cost at the official exchange rate prevailing in the month of surgery yielded a mean of $8,122 (SD $1,296; median $8,143), with the U. S. cost 135–199% higher (roughly 2.4–3.0×), depending on the inflation assumption. This is the most granular approach, which accounts for Naira depreciation over the study period. Under PPP conversion, the Nigerian mean cost was Int$20,422–21,652, comparable in resource-intensity terms to the U. S. cost. The observed differential of $11,430 under the base case exceeded the SD of the Nigerian cohort ($1,979 at the base-case rate) by more than fivefold, indicating that the differential is large relative to variations in patient-level cost.

Both groups were homogeneous in age (*p* = 0.694), but this study had a higher proportion of patients who underwent two-level surgery than the previous cohort (*p* = 0.037). This difference informed the earlier-described subanalysis of SDn, EBL, LOS, and the perioperative surgical complication rate with respect to NOL. EBL, LOS, and the perioperative surgical complication rates were similar across the MIS-TLIF groups (*p* = 0.264, 0.558, and 0.749, respectively), whereas SDn was lower in the previous cohort (*p* < 0.001). Against this backdrop, under the jointly conservative model, the mean cost was statistically lower (*p* < 0.001) in this study (Table 3).

**Table 3:** Comparison of minimally invasive transforaminal interbody fusion between this study and the previous cohort.

Variable	This study*	Past cohort	Test statistic; df; *p*-value	ES/OR; 95% CI	Missing data*, n
Number of cases	65	57	—	—	—
Study duration, years	4	4	—	—	—
Sex, n					
Male	28	17	—	—	0
Female	37	40			
Mean (SD) age, years	60.5 (11.4)	61.1 (N/A)	t = −0.40; 64.0; 0.694	ES = −0.05; −0.29, 0.19	0
Number of levels, n					
1-level surgeries	40	45	χ^2^ = 4.36; 1; 0.037	OR = 0.43; 0.19, 0.96	0
≥ 2-level surgeries	25	12			
Mean (SD) SDn, min	186.2 (56.5)	161.0 (7.6)	t = 3.50; 64.5; < 0.001	ES = 0.61; 0.24, 0.97	2
Mean (SD) EBL, mL	105.0 (65.5)	95.0 (20.0)	t = 1.13; 73.2; 0.264	ES = 0.20; −0.16, 0.56	3
Mean (SD) LOS, days	4.1 (7.1)	3.6 (1)	t = 0.59; 66.9; 0.558	ES = 0.10; −0.26, 0.46	0
Perioperative surgical site complication rate, n (%)	6 (9.2)	4 (7.0)	*p* = 0.749	OR = 1.35; 0.361, 5.04	0
Mean (SD) cost, USD	9,530 (1,979)	20,960 (N/A)	t = −45.9; 62.0; < 0.001	ES = −5.78; −6.80, −4.73	2

Where only the historical mean was available, comparisons are one-sample t-tests and the effect size is a one-sample d expressed in units of the present cohort’s SD; it is not comparable to a two-group d. N/A, Not available; ES, Effect size (Cohen’s d); OR, Odds ratio; CI, Confidence interval; SD, Standard deviation; SDn, Surgery duration; EBL, Estimated blood loss; LOS, Length of hospital stay; USD, U. S. Dollars.

## Discussion

This study reviews the patients, indications, surgical practice, perioperative parameters, perioperative surgical complications, and direct hospital costs for MIS-TLIF performed by the authors in Nigeria, and it compares these results with data from our previous cohort. Some data in this study were missing despite prospective collection. For variables with missingness ≤ 5%, we assumed MCAR because the missingness did not occur systematically, appeared random, and was not associated with other variables (54–56). Additionally, for variables with more than 5% missing data, even in the absence of statistical evidence against MCAR, we assumed MAR and MNAR in the analyses. We believe these steps helped keep the data as robust as possible (54).

The perioperative parameters and surgical complication rates in this study are largely comparable to those reported in multiple previous studies on MIS-TLIF (30, 68–79). Specifically, the two cohorts show statistically similar EBL, LOS, and perioperative surgical complication rates, attesting to the feasibility of performing these surgeries to the same standard in both settings despite differences in development. Another study from the U. S., however, reported perioperative parameters substantially higher than those in our cohort, although its report appears to reflect early experience, given its reference to the early part of its authors’ learning curve for their cases (80). The time interval between the two cohorts raises concerns about potential confounding by the expected increase in surgical proficiency over time. This is because SDn, EBL, LOS, and complication rates in minimally invasive spine surgery, including MIS-TLIF, have been shown to decrease with experience (68, 69, 80, 81). Additionally, the difference between the two cohorts in the proportion of two-level surgeries raises concerns about outcome comparability, as outcomes may worsen with the number of levels operated on (77, 82). Despite these potential effects of experience and increased NOL on the comparison, a statistically significant difference in perioperative parameters occurred only in SDn, which was higher in the current cohort. The higher proportion of two-level surgery in this cohort compared with the previous one may therefore explain the unexpected increase over time. This may be supported by the SDn subanalysis within the Nigerian cohort (two-level SDn was statistically higher than one-level SDn). Another potential explanation for the SDn difference is the senior author’s anecdotal experience that more equipment- and personnel-related delays were observed in our setting than in his U. S. practice. For EBL, LOS, and perioperative surgical complications, the expected improvement with experience may have been offset by the higher proportion of two-level surgeries in the more recent cohort. Visual estimation of blood loss is a longstanding technique that includes assessing the saturation of sponges and drapes, measuring the contents of suction canisters, and estimating the blood present on surfaces in the operating room. Though commonly used globally, it is a subjective method and typically underestimates EBL compared with formula, gravimetric, colorimetric, and pictographic estimation (32, 34). However, like visual estimation, these more objective methods also exhibit intrinsic case-to-case variation (34), raising concerns about reproducibility across them. Although all continuous variables, except SDn, that were compared between the two cohorts were not normally distributed, the use of a parametric test was not only pragmatic. It was also supported by the absence of statistical evidence of asymmetry in this cohort, except for EBL and LOS. The right-skewed distribution of EBL is expected, since perioperative blood loss has a zero floor, with most cases clustering at low volumes, while occasional difficult cases produce a long upper tail. LOS was likewise genuinely right-skewed (*p* < 0.001 even after excluding a single 60-day outlier, showing robustness to the extreme value), as expected for typical short stays with a few prolonged admissions. The mean-based comparison for these two variables should accordingly be interpreted with caution, since the median is more representative for summarizing them.

Both disability and pain improved substantially over the first postoperative year. Pain relief was rapid, reaching a clinically important reduction by 1 month and remaining stable thereafter, whereas disability improved more gradually, with significant gains continuing through six and 12 months. The pain reductions exceeded the NRS MCID at every postoperative time point, and the one-year ODI reduction exceeded the upper bound of the reported MCID range. Rapid pain relief followed by slower functional recovery is the pattern usually reported after lumbar interbody fusion (83, 84).

The main limitation of the PROs was poor follow-up, with about three-quarters of the cohort’s postoperative visits affected, resulting in high, non-monotone missingness. Restricting the analysis to complete cases would have significantly reduced the sample size, but the mixed model, which incorporated all available data under an MAR assumption, and the delta-adjusted tipping-point analysis to assess the influence of missing data provided robustness. The conclusion is that even when every patient with missing data was assumed to have fared substantially worse than predicted, improvements in pain and disability persisted. Additionally, the complete-case analysis and the mixed model showed agreement, supporting the robustness of the findings. Nonetheless, given the degree of missingness, the PRO analyses are exploratory, and their results should be interpreted with caution.

The direct hospital cost of MIS-TLIF in this study is significantly lower than that reported in the U. S. study, despite the highlighted similarities between the two settings and statistically comparable perioperative parameters and surgical complication rates. This cost difference is likely due to lower hospital charges and, probably, lower consumable costs, such as medications and implants, in Nigeria. Because spinal implants and much of the consumable equipment used in Nigeria are imported and effectively priced in USD, that cost component is potentially insulated from exchange-rate effects. This suggests that the cost difference between the two cohorts is driven primarily by locally denominated components (labor, facility, and hospitalization costs). However, the Nigerian Naira fees are typically used to purchase these imported implants and supplies. Overall, despite this cost difference, MIS-TLIF and even some less advanced healthcare options remain out of reach for much of the country due to high poverty rates and inadequate social health insurance coverage (23, 29, 85).

Several limitations, however, affect this cross-country cost comparison. First, nominal exchange-rate conversion does not capture differences in purchasing power: under World Bank PPP conversion factors (47, 48), the Nigerian cohort’s cost is approximately Int$20,400–21,700, comparable to the U. S. cohort’s inflation-adjusted cost. The nominal and PPP results answer different questions, though. The former reflects what the procedure costs in internationally tradeable dollars (relevant, for example, to medical-travel and international payer perspectives). In contrast, the PPP comparison highlights the volume of local resources consumed. Next, cost structures differ. U. S. hospital costs typically include labor, malpractice, regulatory, and administrative burdens, with no direct Nigerian equivalents of comparable scale, so part of the cost difference is likely structural. Third, the two cost constructs are not perfectly aligned: the U. S. figure comprised hospital direct costs (labor, medical supplies including implants, equipment, and general/administrative allocations) and excluded physician professional fees, whereas the Nigerian figure included surgical and anesthesia fees in addition to supplies and admission-through-discharge charges. This asymmetry, like our primary conversion assumptions, works against finding lower Nigerian costs. Also, Nigeria’s multiple-exchange-rate environment during the study period (official, NAFEX/I&E, and parallel rates) means that any single conversion rate is an approximation. Our sensitivity analysis across these rates, under all of which the differential persisted, addresses this. Finally, cost data were non-normally distributed, so means were compared because the reference study reported only means, but means-based comparisons of skewed cost data are sensitive to extreme values. Our assessment of cost skewness, however, demonstrated no statistical evidence of asymmetry.

We believe that the segment of the Nigerian population that often seeks overseas medical treatment, sometimes unsuccessfully due to high healthcare costs, the added costs of travel and lodging, and the absence of family and friend support, may benefit from the availability of MIS-TLIF locally. The expected outcome is a reduction in medical/healthcare tourism and the retention of healthcare expenditure within Nigeria.

As noted in the literature, including our MISS publication (18), access to neurosurgical care in LMICs remains a pressing concern. Our article (18) highlighted critical issues related to starting and sustaining an MISS practice in Nigeria, such as funding for such programs, brain drain, short-term versus sustainable methods for capacity building that lead to brain gain, particularly in the neurosurgical workforce, the need to strengthen health systems in Nigeria, and the utility of advanced techniques like MISS without abandoning routine procedures in developing nations. Interventions from high-income countries (HIC) to low- and middle-income countries, such as twinning and training (86), clinical spine care partnerships (87), and short-term medical missions (88), help improve access to neurosurgical care, but a continuous presence and practice of HIC-trained physicians, as is the case with the senior author, are likely to have a greater impact on direct clinical care and capacity building. This study further supports the feasibility of providing access to much-needed quality surgical care in an LMIC such as Nigeria. We believe that as access to such advanced techniques increases in Nigeria, their costs will diminish, bringing the country closer to the 2030 Global Surgery target of access to safe and affordable surgical care (89).

The comparison of this study’s data with the prior cohort was conducted carefully to minimize chronological bias and other differences arising from spatial and temporal variations between the studies. Both studies had the same lead operator, who used similar operative techniques, surgical equipment, and postoperative management philosophy. Likewise, the operating room environments were essentially similar. This homogeneity was crucial to ensuring the comparison was logical. Despite these efforts, some comparability issues remain, as discussed earlier. Fortuitously, both MIS-TLIF groups had similar age distributions and study durations. We did not compare the sexes, as sex was not expected to affect the outcomes analyzed.

Other limitations of this study include its retrospective design and relatively small sample sizes in both cohorts. Additionally, it is a single-surgeon, single-center experience. MIS-TLIF had not been performed in Nigeria before our series; as more practitioners adopt the technique for their patients, multicenter studies will become possible. The outcomes of such research will make our results generalizable if they corroborate our findings, despite the limitations on reproducibility discussed. Our study’s strengths include a rational comparison between the two studies, sample sizes largely comparable to those in research from developed countries, and sensitivity analyses to ensure robustness. Additionally, obtaining perioperative parameters from sources free of surgeon bias, such as anesthesia and nursing records and patient-reported data, enhanced the reliability of the research.

## Conclusion

MIS-TLIF direct costs in our Nigerian practice are lower than in a developed country, even though perioperative parameters and surgical complication rates are mostly comparable. MISS is feasible in a developing country, yielding good outcomes at reasonable costs.

## Data Availability

The raw data supporting the conclusions of this article will be made available by the authors, without undue reservation.
