# Supplementary material for: Cost comparison of minimally invasive transforaminal lumbar interbody fusion: experience from Nigeria and the United States
Source: Front Public Health. 2026 Sep 28;14:1790794. doi: 10.3389/fpubh.2026.1790794 (PMC13654945; doi:10.3389/fpubh.2026.1790794)
Supplement: Supplementary file 1 [file Table_1.pdf]

Supplementary Materials

Supplementary Methods

Additional model details for PRO sensitivity analyses are presented here. Denominator degrees of freedom were approximated using the Satterthwaite method; a compound-symmetry covariance structure (random intercept) was used rather than an unstructured covariance given the sample size; and estimated marginal means and pairwise contrasts were computed with the emmeans package. For the delta-adjusted sensitivity analysis, age and sex were included as auxiliary variables in the imputation model, the mixed model was refitted to each of the 50 imputed datasets (lme4, emmeans) and pooled by Rubin's rules, and the p-values for the individual baseline contrasts were left unadjusted; a delta of 0 reproduced the primary estimate under MAR (for ODI baseline-to-12 months, −30.4 points versus the primary −28.6 points).

Supplementary Table S1

Skewness of the variables using D'Agostino's skewness test.

Variable	n	Skew (G1)	z	p
Age	65	-0.3563	-1.2536	0.2100
SDn	63	0.4281	1.4732	0.1407
EBL	62	2.2658	5.3977	0.0000
LOS	65	7.7219	9.5732	0.0000
Preoperative NRS	39	-0.0301	-0.0873	0.9304
Preoperative ODI	43	-0.4150	-1.2231	0.2213
One-month NRS	16	0.2126	0.4400	0.6599
One-month ODI	16	0.1191	0.2471	0.8048
Six-month NRS	12	-0.3233	-0.6078	0.5433
Six-month ODI	16	-0.4592	-0.9384	0.3480
12-month NRS	16	-0.0820	-0.1702	0.8649
12-month ODI	17	0.5694	1.1765	0.2394
Cost	63	0.1772	0.6254	0.5317

Supplementary Table S2

Delta-adjusted multiple-imputation sensitivity analysis (m = 50, mice) of the baseline contrasts. Delta added to imputed post-operative values (pessimistic MNAR). Negative estimate = improvement. P-values are unadjusted (each baseline contrast assessed individually). The delta = 0 row is the multiple-imputation estimate under MAR and closely approximates the primary mixed model.

Outcome	Contrast	delta	Estimate	SE	p
ODI	Preoperative to one month	0	−0.092	0.046	0.048
ODI	Preoperative to one month	0.05	−0.054	0.046	0.242

ODI	Preoperative to one month	0.10	−0.016	0.046	0.724
ODI	Preoperative to one month	0.15	+0.021	0.047	0.652
ODI	Preoperative to one month	0.20	+0.059	0.048	0.219
ODI	Preoperative to six months	0	−0.187	0.044	<0.001
ODI	Preoperative to six months	0.05	−0.149	0.044	0.001
ODI	Preoperative to six months	0.10	−0.112	0.045	0.014
ODI	Preoperative to six months	0.15	−0.074	0.045	0.106
ODI	Preoperative to six months	0.20	−0.036	0.046	0.434
ODI	Preoperative to 12 months	0	−0.304	0.045	<0.001
ODI	Preoperative to 12 months	0.05	−0.267	0.045	<0.001
ODI	Preoperative to 12 months	0.10	−0.230	0.046	<0.001
ODI	Preoperative to 12 months	0.15	−0.193	0.046	<0.001
ODI	Preoperative to 12 months	0.20	−0.156	0.047	0.001
NRS	Preoperative to one month	0	−3.974	0.398	<0.001
NRS	Preoperative to one month	1	−3.220	0.402	<0.001
NRS	Preoperative to one month	2	−2.466	0.416	<0.001
NRS	Preoperative to one month	3	−1.712	0.437	<0.001
NRS	Preoperative to six months	0	−3.772	0.648	<0.001
NRS	Preoperative to six months	1	−2.957	0.651	<0.001
NRS	Preoperative to six months	2	−2.141	0.659	0.002
NRS	Preoperative to six months	3	−1.326	0.673	0.052
NRS	Preoperative to 12 months	0	−4.155	0.555	<0.001
NRS	Preoperative to 12 months	1	−3.401	0.558	<0.001
NRS	Preoperative to 12 months	2	−2.647	0.568	<0.001
NRS	Preoperative to 12 months	3	−1.893	0.583	0.001

Footnote: ODI estimates are on the 0–1 scale (multiply by 100 for ODI points); delta for ODI is in ODI points (5 points = 0.05).

Supplementary Table S3

Sensitivity of the MIS-TLIF cost differential to alternative exchange-rate and inflation assumptions. Cell values are the percentage by which the U.S. cost exceeds the Nigerian cost, computed as (US − NG)/NG — i.e., how much more a patient would pay to have the same operation in the U.S. The shaded cell is the manuscript base case. The final row converts each patient's cost at the official exchange rate prevailing in their surgery month.

Nigerian cohort conversion scenario	Nigerian mean cost, USD (95% CI)	No adjustment $19,098	Headline CPI, Dec 2012–Jun 2018 (base case) $20,960	Headline CPI, Dec 2012–Jun 2020 (study midpoint) $21,443	Headline CPI, Jun 2011–Jun 2020 (midpoint-to-midpoint) $21,812	Headline CPI, Dec 2012–May 2022 (study end) $24,313	Medical Care CPI, Dec 2012–Jun 2018 $22,171	Medical Care CPI, Dec 2012–Jun 2020 $23,755
**Official rate, Jul 2018 (IMF; ₦305.90/$; base case)**	**$9,530 (9,032–10,028)**	100.4%	**119.9%**	125.0%	128.9%	155.1%	132.6%	149.3%
Official rate, period average Jun 2018–May 2022 (IMF; ₦355.86/$)	$8,192 (7,764–8,621)	133.1%	155.9%	161.8%	166.3%	196.8%	170.6%	190.0%
NAFEX/I&E window, study end, 23 May 2022 (₦420.33/$)	$6,936 (6,573–7,298)	175.4%	202.2%	209.2%	214.5%	250.6%	219.7%	242.5%
Parallel market, study end, May 2022 (illustrative; ₦604/$)	$4,827 (4,574–5,079)	295.7%	334.3%	344.3%	351.9%	403.7%	359.4%	392.2%
Per-patient conversion at contemporaneous monthly official rate (IMF IFS)	$8,122 (7,796–8,449)	135.1%	158.1%	164.0%	168.5%	199.3%	173.0%	192.5%

Footnote: CPI = Consumer Price Index (U.S. Bureau of Labor Statistics; headline = CPI-U all items, series CUUR0000SA0; Medical Care = series CUUR0000SAM; both not seasonally adjusted). CBN = Central Bank of Nigeria. NAFEX/I&E = Nigerian Autonomous Foreign Exchange Fixing (Investors' & Exporters' window; FMDQ). The parallel-market row is an illustrative upper bound; no official source publishes parallel rates, and its value is drawn from a market aggregator (AbokiFX). Nigerian cohort (n=63 with cost data): mean direct cost

₦2,915,256 (SD ₦605,269; median ₦2,930,850; IQR ₦2,500,000–3,298,500; Shapiro-Wilk $p<0.001$). U.S. cohort mean direct cost: $19,098 (2009–2012). Monthly official rates for the per-patient conversion: IMF International Financial Statistics, series M.NG.ENDE_XDC_USD_RATE (₦305.75–417.00/USD over the surgery dates). 95% CIs are t-based intervals on the Nigerian patient-level costs (mean ± $t_{0.975,62}$ × SE); for fixed-rate rows they equal the NGN interval (₦2,762,821–3,067,691) divided by the rate. They reflect Nigerian-cohort sampling uncertainty only; the U.S. mean is a fixed reference without available variance. The period-average official rate (₦355.86/USD) is the mean of IMF IFS monthly official rates over June 2018–May 2022. Under purchasing-power-parity conversion (World Bank 2020 factors, study midpoint year), the Nigerian mean cost is Int$21,652 (GDP PPP, ₦134.64/Int$) or Int$20,422 (private-consumption PPP, ₦142.75/Int$); PPP values are in international dollars and are not directly comparable to the nominal USD figures in the table.
